# Promoting health equity during the COVID-19 pandemic, United States

**DOI:** 10.2471/BLT.21.286074

**Published:** 2021-12-29

**Authors:** Jazmyn T Moore, Carolina Luna-Pinto, Heidi Cox, Sima Razi, Michael E St. Louis, Jessica N Ricaldi, Leandris Liburd

**Affiliations:** a United States Centers for Disease Control and Prevention, 4770 Buford Highway NE, Atlanta, GA 30341-3717, United States of America.

The United States of America has a diverse population of over 331 million people.^1^ Groups historically identified as racial and ethnic minorities (which make up more than one third of the US population)^1^ have been economically and socially marginalized, leading to lower access to education, health care and financial capital, therefore putting some of these groups at increased risk for poor health outcomes.^2^ The coronavirus disease 2019 (COVID-19) pandemic has amplified existing health inequities; disparities in COVID-19 cases, hospitalizations and deaths, and now vaccination rates, have been identified.^3^^,^^4^ Here, we provide a high-level summary of strategies implemented by the United States Centers for Disease Control and Prevention (CDC) to address COVID-19 inequities impacting racial and ethnic minority groups.

## CDC’s health equity strategy

CDC’s role in the national response to COVID-19 is multifaceted. CDC coordinates activities across the federal government, develops public health guidance and tools, provides technical assistance to jurisdictions, funds jurisdictions and nongovernmental organizations to implement and expand their activities, and serves as a central hub for COVID-19 epidemiologic data.

As COVID-19 disparities emerged, CDC implemented strategies to understand and address them. During May 2020, for the first time in the agency’s history, the Chief Health Equity Officer Unit was established during the emergency response. The unit developed CDC’s COVID-19 Health Equity Strategy^5^ to address the disproportionate impact of COVID-19. The guiding principles of CDC’s COVID-19 Health Equity Strategy are to reduce health disparities, use data-driven approaches, foster meaningful engagement with community institutions and diverse leaders and reduce stigma, including stigma associated with race and ethnicity. CDC implements these principles through four strategies.

The first strategy is to expand the evidence base. COVID-19 data reported by state, tribal, local and territorial health departments provide a national picture of COVID-19. Differences in data collection methods across jurisdictions and limitations of data on race and ethnicity make it difficult to quantify and address disparities.

To help address this challenge, CDC in collaboration with its state, local, tribal and territorial health department partners, added race, ethnicity, social vulnerability (Box 1) and other health equity data to CDC’s publicly available COVID Data Tracker (Box 2).

Box 1 **Social vulnerability index, United States**

The United States Centers for Disease Control and Prevention and Agency for Toxic Substances and Disease Registry Social Vulnerability Index uses 15 United States census variables to help local officials identify communities that may need support before, during or after disasters. The factors considered in developing this vulnerability index include economic data as well as data regarding education, family characteristics, housing language ability, ethnicity and vehicle access. The index values range from 0 (least vulnerable) to 1 (most vulnerable). The index can also be categorized as follows: very low (0.00–0.19), low (0.20–0.39); moderate (0.40–0.59); high (0.60–0.79); very high (0.80–1.00)

Box 2 **Data available in the United States Centers for Disease Control and Prevention COVID-19 data tracker, by health indicator**

Cases: Age, sex, race, ethnicity, pregnancy status, socioeconomic factors, metropolitan status

Hospitalizations: Age, sex, race, ethnicity, pregnancy status, underlying medical conditions

Deaths: Age, sex, race/ethnicity, pregnancy status, socioeconomic status, metropolitan status

Vaccinations: Age, sex, race, ethnicity, disability status, pregnancy status, metropolitan status, social vulnerability

Other: Cases and deaths in correctional and detention facilities, cases and deaths among health care personnel, county-level characteristics (prevalence of underlying medical conditions, pandemic preparedness, percent of population in poverty, percent of population uninsured, average household size, COVID-19 community vulnerability, social vulnerability)

COVID-19: coronavirus disease 2019.

CDC is identifying and promoting innovative ways to analyse race and ethnicity data and has recently published analyses on alternative methods for grouping race and ethnicity to identify disparities^6^ and examining racial subcategories to identify disparities.^7^ CDC has provided over 8 million United States dollars (US$) in funding to 72 projects to support the collection and reporting of relevant COVID-19 data. Detailed race and ethnicity data can help guide ongoing emergency response efforts, direct resources to communities in real time, and inform tailored public health communications. 

The second strategy focusses on expanding programmes and practices to reach populations at increased risk for COVID-19. These populations can also be disproportionately impacted by unintended consequences from mitigation strategies such as business closures that result in lost wages, unemployment, reduced food and housing security, loss of health insurance and others. Focused, culturally responsive efforts can better protect these populations without causing detriment.

CDC has provided technical assistance to health departments across the United States to help reach populations at increased risk for COVID-19. For example, CDC has issued tailored guidance for health departments on contact tracing for refugee, immigrant and migrant populations, strengthened partnerships through continued engagement with American Indian and Alaska Native Tribes, and developed COVID-19 resources in over 30 languages for populations with limited English proficiency. CDC also created and promoted the use of *Health Equity Guiding Principles for Inclusive Communication*,^8^ which emphasize the importance of using non-stigmatizing, bias-free language. CDC has developed a website^9^ that describes best practices for promoting health equity in the context of COVID-19. To improve efforts to reach populations at increased risk for COVID-19, CDC has provided nearly US$ 100 million to over 120 distinct projects. 

The third strategy focusses on supporting essential and frontline workers. The disproportionate impact of COVID-19 on essential and frontline workers was recognized early in the pandemic. Racial and ethnic minority groups are overrepresented in essential work settings^10^ and face a higher risk of being exposed to the severe acute respiratory syndrome coronavirus 2 (SARS-CoV-2) due to the nature of their work, which may require close contact with the public or other workers and often cannot be done from home.^11^ CDC has worked closely with health departments and some employers to help expand programme and practice activities to support essential and frontline workers. CDC has implemented multilingual communication campaigns for agricultural workers, rapid assessments through community-based surveys to better understand COVID-19 risks essential workers face and has provided extensive technical assistance to health-care facilities to improve practices to protect frontline workers. To date, CDC has engaged in nearly 40 projects representing over US$ 13 million to help support essential and frontline workers. 

The fourth strategy concerns the workforce. Diversity exists within and across communities, with variations in history, culture, norms, attitudes, behaviours and lived experiences. The public health workforce should mirror the communities they serve, and CDC remains committed to building a diverse workforce.

To cultivate an inclusive workforce equipped to address the needs of an increasingly diverse population, CDC has funded projects to train new public health staff, including recent graduates, with a special focus on training and hiring from within communities. CDC has also provided funding to increase jurisdictional staffing, and to racial and ethnic minority physicians to increase capacity for implementing COVID-19 prevention, testing, therapeutic and vaccination strategies within their communities. To date, CDC has engaged in 17 projects and provided US$ 7 million in funding towards public health workforce diversity.

## Other crosscutting activities

CDC has also engaged in broader COVID-19 health equity initiatives. From January 2020 through 4 December 2021, 314 teams comprising 1678 CDC staff members have completed deployments to address health equity in 43 states and the District of Columbia, 37 American Indian and Alaska Native Tribes and tribal organizations and six territories and freely-associated states.

In addition to person-hours from CDC staff, and inclusive of what was described previously, CDC has awarded over US$ 5.5 billion supporting COVID-19 health equity activities to various recipients, including American Indian and Alaska Native tribes and tribal organizations, state, territorial and local health departments, community-based organizations, and others. These funds are catalysing data collection and supporting culturally and linguistically responsive approaches to contact tracing, testing, vaccination, and other prevention activities. 

Despite these efforts, some disparities in COVID-19 cases, deaths and vaccination coverage persist. Though racial and ethnic minority groups most affected have varied at different points in time during the pandemic, some groups continue to experience higher number of cases and deaths.^3^^,^^4^ Furthermore, disparities in vaccination rates are also present among racial and ethnic minority groups.^12^ While some efforts to eliminate COVID-19 disparities may result in detectable changes at the community level, the national impact of the efforts of CDC and its partners on cases, deaths and vaccinations received is difficult to estimate.

## Challenges and lessons learnt

The magnitude and duration of the COVID-19 response is far beyond any health emergency CDC has faced previously but it has provided many challenges and opportunities to learn. Though CDC’s Chief Health Equity Officer Unit was established early during the pandemic, integrating a health equity strategy at the very outset of future emergency responses can help ensure that health equity considerations are built into all policies, processes and systems as they are established. While quantifying disparities averted by health equity activities is difficult, early implementation may also help minimize any disproportionate impact on groups that have been marginalized.

Additionally, the usage of epidemiologic data to guide prevention efforts has been hindered by a lack of complete demographic data. Implementing strategies to capture more complete demographic data can help improve efforts to reach underrepresented populations, including racial and ethnic minority groups, people with disabilities, people experiencing homelessness, sexual and gender minority groups, and other groups that have been marginalized.

COVID-19 mitigation activities have had unintended consequences that, in some cases, have deepened existing inequities. Examining unintended consequences caused by mitigation policies and learning from them can help inform community-driven public health activities in the future. Engaging in community outreach and creating opportunities to listen to community members can help build trust and strong partnerships and drive culturally and linguistically responsive activities.

While substantial investments in training the public health workforce have been made through various funding mechanisms, a fundamental need for the workforce to represent the people in the communities it services still exists. Broader efforts at all levels (including schools and health departments, among others) are needed to hire and train public health workers from diverse backgrounds.

The COVID-19 pandemic has highlighted the need to apply a health equity lens while implementing disease prevention and control strategies. Through the implementation of its health equity strategy, CDC embedded health equity considerations more consistently and broadly than in past major emergency responses. Ultimately, health disparities will persist until structural issues contributing to inequity, including the social determinants of health, are addressed. Though much work is needed, with continued financial investment, community and partner engagement, national policies aimed at structural factors that influence health, and CDC's commitment to achieving health equity, the goal of equity can still be achieved.
